# Exercise dosage to facilitate the recovery of balance, walking, and quality of life after stroke

**DOI:** 10.4102/sajp.v79i1.1846

**Published:** 2023-02-10

**Authors:** Elogni R. Amanzonwé, Lisa Tedesco Triccas, Léopold Codjo, Dominique Hansen, Peter Feys, Oyéné Kossi

**Affiliations:** 1 Unit of NeuroRehabilitation, Department of Neurology NeuroRehabilitation, University of Parakou, Parakou, Benin; 2 REVAL, Rehabilitation Research Center, Faculty of Rehabilitation Sciences, Hasselt University, Hasselt, Belgium; 3 Department of Cardiology, Faculty of Medicine, University of Parakou, Parakou, Benin; 4 Heart Centre Hasselt, Jessa Hospital, Hasselt, Belgium; 5 Unit of NeuroRehabilitation, Department of Neurology NeuroRehabilitation, University Hospital of Parakou, Parakou, Benin; 6 ENATSE, National School of Public Health and Epidemiology, University of Parakou, Parakou, Benin

**Keywords:** balance, exercise therapy, quality of life, stroke, walking ability

## Abstract

**Background:**

Although aerobic training (AT) and resistance training (RT) are recommended after stroke, the optimal dosage of these interventions and their effectiveness on balance, walking capacity, and quality of life (QoL) remain conflicting.

**Objectives:**

Our study aimed to quantify the effects of different modes, dosages and settings of exercise therapy on balance, walking capacity, and QoL in stroke survivors.

**Method:**

PubMed, CINHAL, and Hinari databases were searched for randomised controlled trials (RCTs) evaluating the effects of AT and RT on balance, walking, and QoL in stroke survivors. The treatment effect was computed by the standard mean differences (SMDs).

**Results:**

Twenty-eight trials (*n* = 1571 participants) were included. Aerobic training and RT interventions were ineffective on balance. Aerobic training interventions were the most effective in improving walking capacity (SMD = 0.37 [0.02, 0.71], *p* = 0.04). For walking, capacity, a higher dosage (duration ≥ 120 min/week; intensity ≥ 60% heart rate reserve) of AT interventions demonstrated a significantly greater effect (SMD = 0.58 [0.12, 1.04], *p* = 0.01). Combined AT and RT improved QoL (SMD = 0.56 [0.12, 0.98], *p* = 0.01). Hospital located rehabilitation setting was effective for improving walking capacity (SMD = 0.57 [0.06, 1.09], *p* = 0.03) compared with home and/or community and laboratory settings.

**Conclusion:**

Our findings showed that neither AT nor RT have a significant effect on balance. However, AT executed in hospital-located settings with a higher dose is a more effective strategy to facilitate walking capacity in chronic stroke. In contrast, combined AT and RT is beneficial for improving QoL.

**Clinical implications:**

A high dosage of aerobic exercise, duration ≥ 120 min/week; intensity ≥ 60% heart rate reserve is beneficial for improving walking capacity.

## Introduction

Balance disorders are recurrent problems in stroke survivors, which could directly affect walking capacity, leading to a poor quality of life (QoL) (Kossi et al. 2021; Schmid et al. 2013). The main causes of poor balance after a stroke are impaired muscle coordination and loss of sensation on the affected side, especially in the legs and trunk (Aries et al. 2022; Gath et al. 2021). For patients with stroke and their families, achieving independence in activities of daily living (ADL) is often the primary concern (Saulle & Schambra 2016). Recovery of walking ability is particularly important for stroke survivors because it is often essential for social participation (Adoukonou et al. 2018; Nindorera et al. 2022; Preston et al. 2011).

To facilitate recovery after a stroke, the implementation of rehabilitation is promoted. Improving walking ability remains a challenge for stroke rehabilitation practitioners to help stroke survivors improve their QoL (Corbetta, Imeri & Gatti 2015). Recent guidelines for rehabilitation after a stroke suggest task-specific training exercises (Pogrebnoy & Dennett 2020). As a consequence, the role of structured exercise-based rehabilitation in post-stroke recovery has been highlighted, where various modes of exercise therapy are used (Pogrebnoy & Dennett 2020).

Aerobic training (AT) is physical activity that implicates the body’s large muscle activity (e.g. graded walking, stationary cycle ergometry) in a rhythmic manner for a sustained period (Ambrosetti et al. 2020). There is evidence that AT improves walking performance, but conflicting evidence regarding balance and QoL in post-stroke (Han et al. 2017). In contrast, resistance training (RT) is a form of exercise that aims to increase muscular strength, endurance and power (Han et al. 2017). There is conflicting evidence that RT results in increases in balance, walking performances and QoL (Han et al. 2017; Saunders et al. 2016).

Some previous meta-analyses reported that aerobic and resistance exercises could improve balance and mobility in patients who recover from a stroke (Lee & Stone 2020; Pogrebnoy & Dennett 2020; Saunders et al. 2020). However, the optimal mode (aerobic vs. resistance or both) and dose (volume, intensity) of exercise required to induce the most significant clinical benefits in balance, walking capacity, and QoL after stroke remain to be determined.

None of the previous meta-analyses on this topic investigated the effect of intervention settings (hospital, home and/or community or research laboratory) and exercise modes on balance, walking capacity and QoL. This latter exploration might influence the level of patient involvement in exercise programmes and, therefore, the results. Our systematic review and meta-analysis aimed to quantify the effects of different modes and dosages of physical exercise therapy on balance, walking and QoL, considering the different settings in which these programmes were executed.

## Methods

Our study was performed following a protocol previously registered in the prospective international register of systematic reviews, PROSPERO (https://www.crd.york.ac.uk/PROSPERO/; registration number: CRD42020202990).

### Data sources and search strategy

Two authors (E.R.A. and L.T.T.) systematically searched PubMed, CINAHL and Hinari databases from the date of inception of the databases until 15 September 2021. They restricted searches to articles involving human participants and written in English and French. The search strategy used the following keywords to query all the databases: exercise training, physical therapy, balance, postural balance, postural control, walking, QoL, stroke and cardiovascular accident. A third reviewer (O.K.) was consulted to resolve conflicts during the title, abstract screening and full-text evaluation. Additional manual searches included conference abstracts, bibliographies of candidate studies and recent systematic reviews for a comprehensive literature search.

### Data extraction and analysis

Two authors (E.R.A. and L.T.T.) independently extracted data from eligible studies using a data extraction spreadsheet with predetermined content. Data included general information on the publications (first author’s name and year of publication), characteristics of the studies (sample size, randomisation and blinding), participants (age, gender and time since stroke), mode of interventions (AT, RT, or combination), the content of the interventions (modality, frequency, intensity, duration and sessions length), setting (hospital-based, home and/or community-based, or research laboratory-based) and outcomes (observation time points, measurement tools and follow-up). All data and other materials used in the review are available upon request.

### Eligibility criteria

The inclusion criteria for study selection were: (1) randomised controlled trials (RCTs) that involved adult patients (age ≥ 18 years); (2) diagnosed with a stroke; (3) executed structured exercise interventions based on AT, RT, or combination; (4) compared experimental groups with active control groups where participants received treatment such as conventional physiotherapy, or a home exercise programme; (5) reported outcome measures to evaluate balance and/or walking capacity and/or QoL.

Studies that included an additional intervention, such as conventional physiotherapy at the same period were excluded from examining the superiority of exercise over other physiotherapy interventions.

### Quality assessment

The authors used the Physiotherapy Evidence Database Scale (PEDro) (Blobaum 2006) to assess the methodological quality of included RCTs. The risk of bias in selected studies was analysed using the Cochrane risk of bias assessment tool. The risk of bias and quality of evidence were assessed by two authors independently (E.R.A. and L.T.T.). Disagreements between the two review authors regarding the methodological quality or risk of bias of some studies were resolved by discussion, with the participation of a third author (O.K.) if necessary.

### Data synthesis and statistical analysis

Data synthesis was performed with Review Manager software (Version 5.3) under the random effects model in which a *p* < 0.05 (two-tailed) was considered significant. The treatment effect was measured by computing standard mean differences (SMDs) with 95% CIs. The authors performed subgroup analyses by stratifying results by exercise modes, volume and intensity of AT interventions and intervention provision settings. The sensitivity analysis was carried out by excluding studies with a high risk of bias and those with poor methodological quality to ensure the robustness of the data. A pre-set cut-off point of ≥ 50% was used to select trials for the sensitivity analysis (Nduwimana et al. 2020).

### Ethical considerations

This systematic review and meta-analysis did not require formal ethical clearance because all data were obtained from publicly available sources and were analysed anonymously.

## Results

### Study selection

The authors identified 3245 records of possible interest in the electronic database searches. After removing duplicates, screening titles, abstracts, and reviewing full texts, 28 RCTs were eligible for qualitative synthesis and met the study’s inclusion criteria (Figure 1). Ultimately, 25 trials reported sufficient data to be included in the quantitative analysis yielding 1571 participants with sample sizes ranging from *n* = 12 to *n* = 128.

**FIGURE 1:**
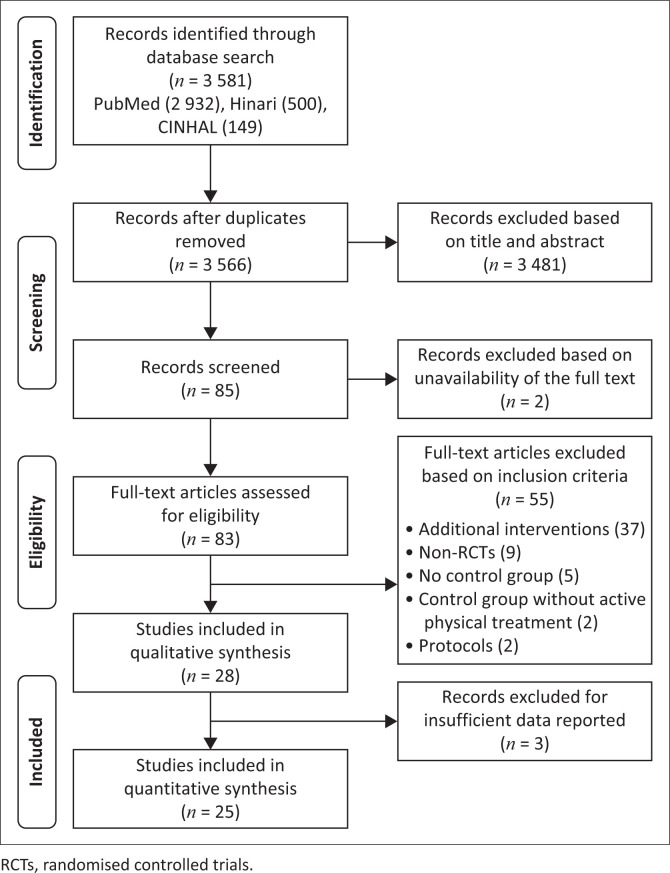
Preferred Reporting Items for Systematic Reviews and Meta-Analyses (PRISMA) flow diagram of inclusion.

### Study characteristics

Descriptive study characteristics of the 28 included studies are shown in Table 1. It was found that 16 RCTs examined AT, 2 RCTs examined the effectiveness of RT, and 10 RCTs examined the effect of combining AT and RT. All studies included chronic stroke survivors except one (Mead et al. 2007), with the mean time post-stroke ranging from 4.9 to 99.2 months across all studies. Fourteen trials were conducted as hospital-based, six as home or community-based, and six trials as research laboratory-based. Twenty-five trials were considered for quantitative analyses (meta-analysis) because three trials did not report sufficient data (Elsner et al. 2020; Mead et al. 2007; Severinsen et al. 2014).

**TABLE 1:** Overview of included studies.

Study ID	Participants	Exercise protocol	Control group	Outcomes (Instruments)§
Ada et al. 2003	*n* = 27 Age: (Exp = 66 ± 11; Con = 66 ± 11) Time since stroke: (Exp = 28 ± 17; Con = 26 ± 20) months Setting: Community	Mode: 4 weeks of treadmill walking + Overground walking Intensity: 80% – 50% HRR (decreasing by 10% each week) Duration: 45 min Frequency: 3× per week (12 sessions)	Placebo, home exercise programme	Walking capacity (distance over 6 min): + (*p* < 0.001) QoL (Sickness Impact Profile): 0
Aguiar et al. 2020	*n* = 22 Age: (Exp = 52 ± 11; Con = 48 ± 10) Time since stroke: (Exp = 51 ± 68; Con = 44 ± 26) months Setting: research laboratory	Mode: 12 weeks of aerobic treadmill training Intensity: 60% – 80% HRR Duration: 40 min Frequency: 3× per week (36 sessions)	Comfortable overground walking	Walking capacity (6-min walk test): 0 QoL (Stroke-Specific QoL scale): + (*p* = 0.017)
Bonnyaud et al. 2014	*n* = 56 Age: (Exp = 49.7 ± 13.5; Con= 68.7 ± 6.1) Time since stroke: (Exp = 69.6 ± 55.2; Con = 74.4 ± 105.6) Setting: NR	Mode: a single overground walking training session Intensity: comfortable gait speed Duration: 20 min Frequency:1× per week	Treadmill walking training	Balance (Timed Up and Go): 0
Clark and Patten 2013	*n* = 34 Age: (Exp = 63.2 ± 10.6; Con= 59.7 ± 10.9) Time since stroke: (Exp = 13.3 ± 4.9; Con = 12.8 ± 4.7) months Setting: NR	Mode: 5 weeks of eccentric RT group + 3 weeks of gait training Intensity: 3–4 sets of 10 repetitions (isokinetic dynamometer) for RT and maximal speed at interval training with short bouts (75–150 s) for gait training Duration: 90 min Frequency: 3× per weeks (15 sessions RT+ 9 sessions AT)	concentric RT group + gait training	Balance (Self-selected and fast walking speeds): + (*p* = 0.04)
Combs-Miller et al. 2014	*n* = 20 Age: (Exp = 56.20 ± 7.61; Con = 57 ± 11) Time since stroke: (Exp = 62.30 ± 48.64; Con = 60 ± 51.68) months Setting: Community	Mode: 2 weeks of BWSTT Intensity: Fairly light to somewhat hard on RPE Duration: 30 min Frequency: 5× per week (10 sessions)	Overground walking training	Walking capacity (6-min walk): 0
Danks et al. 2016	*n* = 27 Age: (Exp = 59.1 ± 8.7; Con = 58.2 ± 12.4) Time since stroke: (Exp = 29.4 ± 21.4; Con = 50.8 ± 44.1) Setting: research laboratory	Mode: 12 weeks of fast walking training (treadmill and overground walking training) plus a step activity programme Intensity: 80% HRR Duration: 40 min Frequency: 1× per week (12 sessions)	Fast walking training alone	Walking capacity (6-min walk): + (*p* = 0.018)
Dean et al. 2000	*n* = 12 Age: (Exp = 66.2 ± 7.7; Con = 62.3 ± 6.6) Time since stroke: (Exp = 27.6 ± 8.4; Con = 15.6 ± 10.8) months Setting: Hospital	Mode: 4 weeks of affected lower limb strengthening and functional tasks Intensity: NR Duration: 60 min Frequency: 3× per week (12 sessions)	Sham upper-limb tasks	Walking capacity (6-min walk test): + (*p* < 0.05)
Drużbicki et al. 2016	*n* = 46 Age: (Exp = 59.9 ± 11.4; Con = 61.5 ± 10.8) Time since stroke: (Exp = 46.1 ± 43.0; Con = 40.2 ± 40.8) months Setting: Hospital	Mode: 2 weeks of treadmill walking with visual feedback Intensity: NR Duration: 30 min Frequency: 5× per week (10 sessions)	Treadmill without biofeedback	Balance (Up & Go test):0
Elsner et al. 2020†	*n* = 12 Age: (Exp = 68.7 ± 11; Con = 67.8 ± 12.3) Time since stroke: (Exp = 34.7 ± 20.1; Con = 99.2 ± 88.5) months Setting: Hospital	Mode: 4 weeks of overground Gait training with rhythmic auditory stimulation (RAS) Intensity: NR Duration: 30 min Frequency: 3× per week (12 sessions)	Overground Gait training without RAS	Balance (Berg Balance Scale): 0 Walking capacity (6-min walk test): 0
Gama et al. 2017	*n* = 28 Age: (Exp = 58.7 ± 8.4; Con = 57.7 ± 10.1) Time since stroke: (Exp = 60.2 ± 55.4; Con = 53.8 ± 42.2) months Setting: research laboratory.	Mode: 6 weeks of BWSTT Intensity: comfortable speed Duration: 45 min Frequency: 3× per week (18 sessions)	Overground Gait training with body weight support	Walking capacity (6-min walk test): + (*p* = 0.001)
Globas et al. 2012	*n* = 36 Age: (Exp = 68.6 ± 6.7; 68.7 ± 6.1) Time since stroke: (Exp = 60.2 ± 46.6; Con = 70 ± 67.4) months Setting: Hospital	Mode: 3 months of progressive graded high-intensity aerobic treadmill exercise Intensity: 60% – 80% HRR (started at 40% – 50% HRR) Duration: 30–50 min Frequency: 3× per week 3 sessions /week (total of 39 sessions)	Conventional care physiotherapy	Balance (Berg Balance Scale): + (*p* < 0.05) Walking capacity (6-min walk test): + (*p* < 0.001) QoL (mental subscore of 12-Item Short Form Health Survey): + (*p* < 0.01)
Gordon et al. 2013	*n* = 128 Age: (Exp = 63.4 ± 9.4; Con = 64.9 ± 11.1) Time since stroke: (Exp = 12.8 ± 3.6; Con = 11.8 ± 3.6) months Setting: Hospital	Mode: 12 weeks of overground brisk walking training Intensity: 60% – 80% HRR Duration: 30 min Frequency: 3× per week (36 sessions)	Massage	Walking capacity (6-min walk test): + (*p* < 0.001) QoL (36-Item Short Form Health Survey): 0
Ivey et al. 2015	*n* = 34 Age: (Exp = 61 ± 1.6; Con = 63 ± 2.4) Time since stroke: (Exp = 41 ± 12; Con = 37 ± 14) months Setting: Hospital	Mode: 24 weeks of higher-intensity treadmill training Intensity: 80% – 85% HRR (started at 40% – 50%) Duration: 30 min Frequency: NR	Lower-intensity treadmill training	Walking capacity (6-min walk distance): 0
Ivey et al. 2017	*n* = 64 Age: (Exp = 57 ± 14; Con = 55 ± 9) Time since stroke: (Exp = 60 ± 48; Con = 72 ± 60) months Setting: Hospital	Mode: 3 months of pneumatic resistance machines (leg press, leg extension and leg curl) Intensity: 20 × 2 × 3 repetitions Duration: 45 min Frequency: 3× per week (36 sessions)	Attention-matched stretch	Walking capacity (6-min walk distance): + (*p* < 0.05)
Janssen et al. 2008	*n* = 12 Age: (Exp = 54.2 ± 10.7; Con = 55.3 ± 10.4) Time since stroke: (Exp = 12.3 ± 5.4; Con = 18.3 ± 9.9) months Setting: Hospital	Mode: 6 weeks of cycling exercise with Electric Stimulation evoking muscle contractions Intensity: HRpeak Duration: 25–30 min Frequency: 2× per week (12 sessions)	Cycling exercise with electric stimulation not evoking muscle contractions	Balance (Berg Balance Scale): 0 Walking capacity (6-min walk distance): + (*p* = 0.035).
Jin et al. 2013	*n* = 128 Age: (Exp = 57.6 ± 6.6; Con = 56.3 ± 6.5) Time since stroke: (Exp = 18.7 ± 5.2; Con = 17.9 ± 4.8) months Setting: Hospital	Mode: 12 weeks of progressive aerobic cycling training Intensity: 50% – 70% HRR (started at 40% – 50% HRR) Duration: 40 min Frequency: 5× per week (60 sessions)	Conventional therapy	Balance (Berg Balance Scale): 0 Walking capacity (6-min walking distance): + (*p* < 0.001)
Lamberti et al. 2017	*n* = 35 Age: (Exp = 69 ± 9; Con = 67 ± 10) Time since stroke: (Exp = 34 ± 46; Con = 40 ± 51) months Setting: Community	Mode: 8 weeks of overground intermittent walking and muscle power training Intensity: Week 1–4: 90 ± 4 step/min, week 5–8: 74.5 ± 3.5 step/min (AT) and week 5–8: 40% – 50% 1RM (RT) Duration: 60 min Frequency: 3× per week (24 sessions)	Treadmill walking and strength training	Balance (Berg Balance Scale): 0 Walking capacity (6-min walking distance): + (*p* = 0.009) QoL (SF36 physical activity domain): + (*p* = 0.012)
Lee et al. 2008‡	*n* = 48 Age: (Exp = 63.5 ± 10.1; Con = 65.3 ± 6) Time since stroke: (Exp = 53.2 ± 35.5; Con = 65.8 ± 42.3) months Setting: research laboratory	Mode: 10–12 weeks of aerobic cycle training and progressive RT (pneumatic resistance, weights, isometric training) Intensity (cycling: 50% – 70% VO2peak; PRT: 50% – 80% of 1RM; 2 × 8 repetitions unilaterally) Duration: 30 × 2 = 60 min Frequency: 3× per week (30 sessions)	Sham cycling and sham progressive RT	Walking capacity (6-min walking test): 0 QoL (36-Item Short Form Health Survey): 0
Lee et al. 2015	*n* = 26 Age: (Exp = 64 ± 7.4; Con = 63 ± 5.5) Time since stroke: (Exp = 71.7 ± 39.9; Con = 69.9 ± 30.1) months Setting: Community	Mode: 16 weeks of Combined aerobic (cycle, walking) and resistance exercise (elastic bands) Intensity (aerobic: 50% – 70% HRR; resistance: 2–3 × 10–15 repetitions; RPE_6–20_ = 11–16) Duration: 60 min Frequency: 3× per week (48 sessions)	Usual care	Walking capacity (6-min walk test): + (*p* < 0.001)
Lo et al. 2012	*n* = 20 Age: (Exp = 47.6 ± 3.3; Con = 51.6 ± 3.4) Time since stroke: (Exp = 25.54 ±12.95; Con = 29.64 ± 10.36) months Setting: Hospital	Mode: A single functional electrical stimulation cycling training Intensity 45 rpm Duration: 20 min Frequency: 1× per week	Cycling	Balance (Smart Balance Master system): + forward direction (*p* = 0.008) and directional control (*p* = 0.028)
Lund et al. 2018†	*n* = 43 Age: (Exp = 67.5 ± 8.4; Con = 66.4 ± 8.8) Time since stroke: (Exp = 18.3 ± 6.5; Con = 17.6 ± 7.7) months Setting: Hospital	Mode: 12 weeks of aerobic training on a cycle ergometer, RT of the lower extremities (leg press, elastic bands) Intensity (cycling: 70% HRR, RPE_6–20_ = 14–16; resistance: 3 × 8 repetitions unilaterally, 80% 1RM) Duration: 36 min Frequency: 3× per week (36 sessions)	Sham training of upper extremities	Balance (Berg Balance Scale): 0 Walking capacity (6-min walk test): 0
Macko et al. 2005	*n* = 61 Age: (Exp = 63 ± 10; Con = 64 ±8) Time since stroke: (Exp = 35 ± 29; Con = 39 ± 59) months Setting: Hospital	Mode: 6 months of treadmill walking Intensity: 60% – 70% HRR (started at 40% – 50% HRR) Duration: 40 min Frequency: 3× per week (36 sessions)	Usual care	Walking capacity (6-min walk test): + (*p* < 0.02)
Marzolini et al. 2018	*n* = 64 Age: (Exp = 61.7 ± 10.0; Con= 65.6 ± 13.2) Time since stroke: (Exp = 14.6 ± 15.5; Con = 9.3±5.7) months Setting: Community	Mode: 24 weeks of aerobic (cycling) and RT Intensity: 60% – 80% HRR (AT) and 50% – 70% 1RM (RT) Duration: 60 min Frequency: 3× per week (AT) plus 2×/week (RT)	Aerobic training	Walking capacity (6-min walk test): 0
Mead et al. 2007	*n* = 66 Age: (Exp = 72.0 ± 10.4; Con = 71.7 ± 9.6) Time since stroke: (Exp = 5.7; Con = 4.9) months Setting: Hospital	Mode: 12 weeks of progressive endurance (cycle, shuttle walking) and resistance (elastic bands, pole-lifting and sit-to-stand exercise) Intensity: (endurance exercise: RPE_6–20_ = 13–16; resistance: 4 × 3 × 10–15 repetitions) Duration: 30–60 min Frequency: 3× per week (36 sessions)	Relaxation	QoL (36-Item Short Form Health Survey): + (*p* = 0.002) for role-physical item
Olney et al. 2006	*n* = 72 Age: (Exp = 63.5 ± 12.0; Con= 65.8 ± 11.6) Time since stroke: (Exp = 49.2 ± 52.8; Con = 40.8 ± 46.8) months Setting: Community	Mode: 10-week supervised strengthening and conditioning programme Intensity: 50% – 70% HRR (AT) Duration: 90 min Frequency: 3× per week (30 sessions)	1-week supervised instruction programme followed by 9-week unsupervised home	Walking capacity (6-min walk test): 0 QoL (SF-36 Physical Component): + (*p* < 0.01)
Ouellette et al. 2004	*n* = 42 Age: (Exp = 65.8 ± 2.5; Con = 66.1 ± 2.1) Time since stroke: (Exp = 31.8 ± 3.3; Con = 25.6 ± 4.0) months Setting: research laboratory	Mode: 12 weeks of high-intensity progressive RT (pneumatic resistance equipment, weight stack-pulley system) Intensity: 3 × 8–10 repetitions at 70% of 1RM Duration: NR Frequency: 3× per week (36 sessions)	Upper extremity stretching	Walking capacity (6-min walk): + (*p* < 0.001)
Quaney et al. 2009	*n* = 38 Age: (Exp = 64.1 ± 12.3; Con = 58.9 ± 14.6) Time since stroke: (Exp = 61.3 ± 42.3; Con = 61.3 ± 42.3) months Setting: research laboratory	Mode: 8 weeks of progressive aerobic bicycle exercise Intensity: 40% – 70% HRR Duration: 45 min Frequency: 3× per week (24 sessions)	Stretching exercise	Balance (Berg Balance Scale): 0
Severinsen et al. 2014†	*n* = 43 Age: (Exp = 68.5; Con = 66) Time since stroke: (Exp = 16.5; Con = 16) months Setting: Hospital	Mode: 12 weeks of aerobic (cycle) with progressive RT (machines) Intensity: (cycle:75% HRR, RPE **_6–20_** = 14–16; resistance: 3 × 8 repetitions, 80% of 1RM) Duration: 60 min Frequency: 3× per week (36 sessions)	Sham training	Walking capacity (6-min walk distance): 0

Exp, experimental group; Con, control group; HRR, heart rate reserve; NR, not reported; RM, repetition maximal; RPE, rating of perceived exertion; AT, aerobic training; RT, resistance training; QoL, quality of life; BWSTT, body weight support treadmill training.

† , Insufficient data reported.

‡ , Insufficient data reported about quality of life.

§ , + indicates significant between-group difference; 0 = no difference between-group.

### Methodological quality and risk of bias assessment

Of the 28 included trials for qualitative synthesis, 25 (89.3%) were of good methodological quality (Ada et al. 2003; Aguiar et al. 2020; Bonnyaud et al. 2014; Clark & Patten 2013; Combs-Miller et al. 2014; Danks, Pohlig & Reisman 2016; Drużbicki et al. 2016; Elsner et al. 2020; Gama et al. 2017; Globas et al. 2012; Gordon, Wilks & McCaw-Binns 2013; Ivey et al. 2015, 2017; Janssen et al. 2008; Jin et al. 2013; Lamberti et al. 2017; Lee et al. 2008, 2015; Lund et al. 2018; Marzolini et al. 2018; Mead et al. 2007; Olney et al. 2006; Ouellette et al. 2004; Quaney et al. 2009; Severinsen et al. 2014), and the remaining trials were of fair quality (Dean, Richards & Malouin 2000; Lo et al. 2012; Macko et al. 2005).

The majority of the included studies (78.6%) were carried out with a low risk (Cochrane risk of bias score > 3) of bias (Ada et al. 2003; Aguiar et al. 2020; Bonnyaud et al. 2014; Clark & Patten 2013; Combs-Miller et al. 2014; Danks et al. 2016; Drużbicki et al. 2016; Elsner et al. 2020; Gama et al. 2017; Globas et al. 2012; Gordon et al. 2013; Ivey et al. 2015, 2017; Jin et al. 2013; Lamberti et al. 2017; Lee et al. 2008, 2015; Marzolini et al. 2018; Mead et al. 2007; Olney et al. 2006; Quaney et al. 2009; Severinsen et al. 2014), and the remaining trials presented a risk of bias score less than three (Dean et al. 2000; Janssen et al. 2008; Lo et al. 2012; Lund et al. 2018; Macko et al. 2005; Ouellette et al. 2004).

### Post-intervention effects of exercise modes

Nine trials involving 405 participants reported the effects of exercise modes on balance (Appendix 1, Figure 1-A1). The analysis showed that neither AT alone nor AT combined with RT significantly improved balance.

**FIGURE 2:**
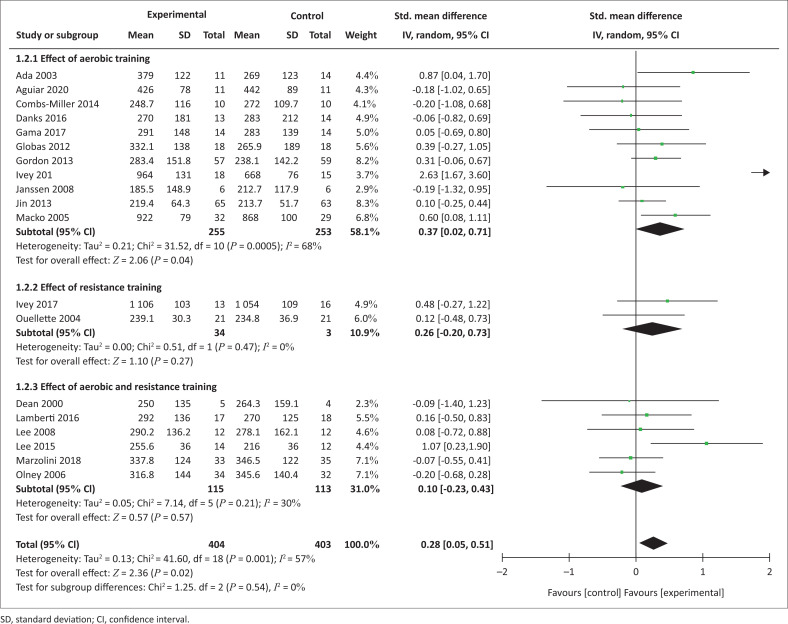
Effect of exercise modes on walking capacity.

Nineteen trials yielding 807 participants reported post-intervention effects of exercise modes on walking capacity (Figure 2). The overall analysis showed a significant effect in favour of experimental interventions (SMD = 0.28 [0.05, 0.51], *p* = 0.02). In the subgroup analysis, AT interventions were more effective than the control interventions on walking capacity, while RT or the combination of AT and RT were not.

**FIGURE 3:**
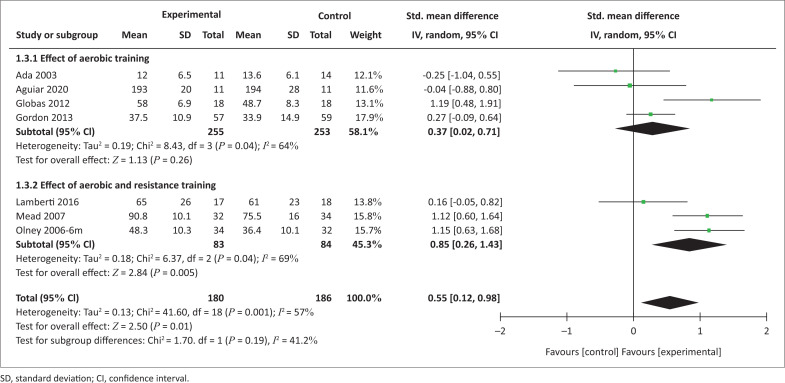
Effect of interventions on quality of life.

Seven trials yielding 366 participants reported post-intervention effects of exercise modes on QoL (Figure 3). The overall analysis demonstrated a significant effect in favour of experimental interventions (SMD = 0.56 [0.12, 0.98], *p* = 0.01). In the subgroup analysis, AT combined with RT was more effective compared with the control interventions on QoL, while AT alone was not.

### Subgroup analyses

**FIGURE 4:**
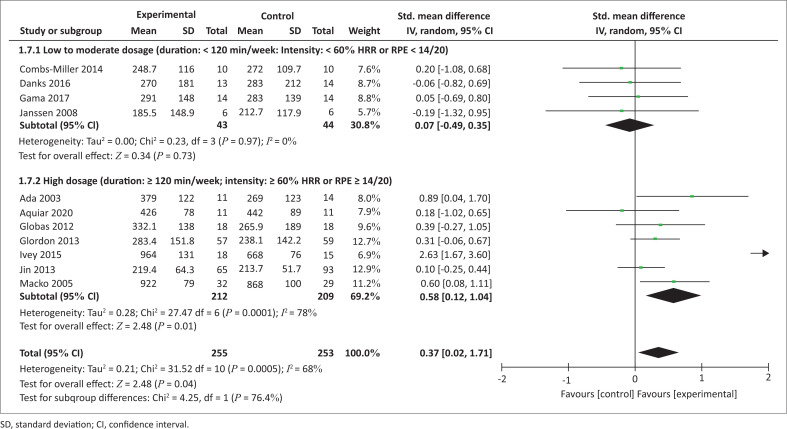
Effect of aerobic training dosage on walking capacity.

The authors compared the effect of AT interventions on walking capacity according to the dosage of interventions (Figure 4). With a duration of at least 120 min of exercise per week and an intensity of at least 60% of heart rate reserve (HRR) or rating of perceived exertion (RPE) above 14/60 per session (high dosage), AT interventions were more effective compared with the control interventions (SMD = 0.58 [0.12, 1.04], *p* = 0.01) while a lower to moderate dosage of AT interventions (< 120 min/week and < 60% HRR or RPE < 14/20) were not.

### Effects of health service setting

**FIGURE 5:**
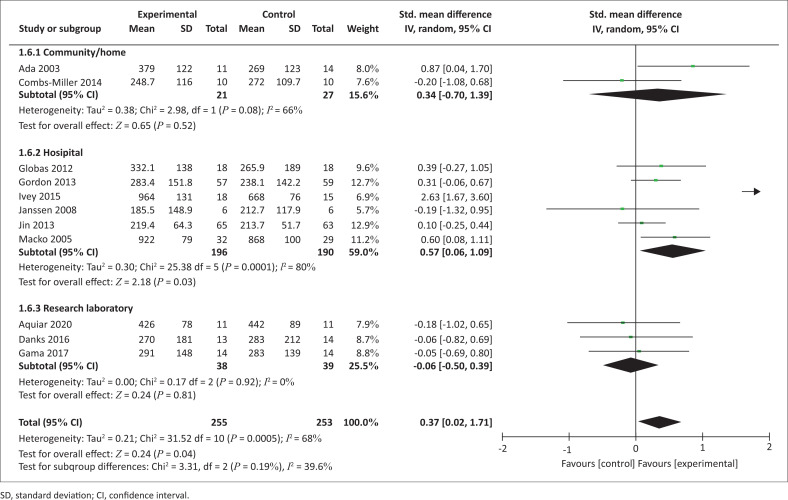
Effect of aerobic training setting of provision on walking capacity.

Eleven trials yielding 508 participants reported the effect of AT setting provision on walking capacity (Figure 5). Analyses showed an improvement in the walking capacity in favour of interventions executed in the hospital setting (SMD = 0.57 [0.06, 1.09], *p* = 0.03).

### Sensitivity analysis

**FIGURE 6:**
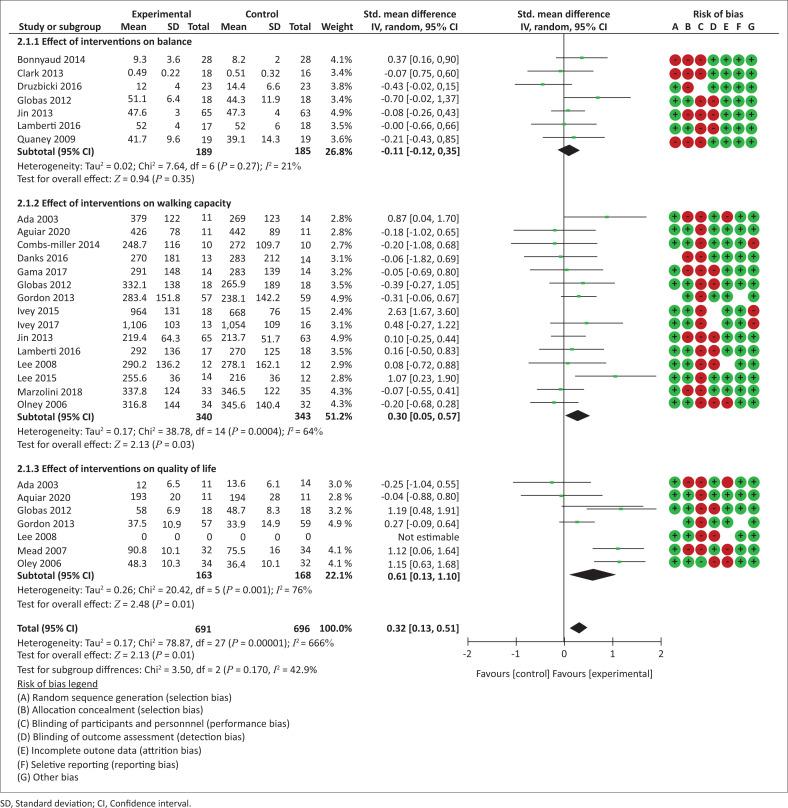
The overall effect of interventions on balance, walking capacity and quality of life after sensitivity analysis.

Treatment significance effects remained similar across different analyses involving only trials with PEDro scores ≥ 6 and Cochrane risk of bias scores > 3 (Figure 6).

## Discussion

Our meta-analysis aimed to quantify the effects of different modes, dosages and settings of exercise therapy on balance, walking capacity, and QoL in stroke survivors. Our findings showed that higher dosages of AT interventions executed in the hospital setting effectively improved walking capacity. At the same time, AT plus RT was more effective in improving QoL in chronic stroke survivors.

The AT is the most effective exercise mode when collectively evaluating all primary outcomes. However, the authors showed that exercise interventions (AT, RT, or AT plus RT) were ineffective in facilitating improved balance in chronic stroke. Recent meta-analyses have suggested that AT (Gelaw et al. 2019) and RT (Veldema & Jansen 2020; Wist, Clivaz & Sattelmayer 2016) had no significant advantage in improving balance. Saunders et al. (2020) reported low to moderate certainty evidence for improving balance through exercise therapy. Our review did not include studies that involved patients receiving other supplementary treatments, such as usual care. This could explain the lack of improvement in balance by exercise therapy in our study. As a result, when balance is a significant issue in some patients who recover from stroke, other treatment options should be considered. A recent systematic review and meta-analysis (Hugues et al. 2019) reported that functional task training associated with musculoskeletal and cardiopulmonary and sensory interventions seems to improve balance and postural stability, respectively.

Our finding that AT improved walking capacity is similar to previous studies. Two systematic reviews and meta-analyses reported that cycling effectively improves walking capacity (Shariat et al. 2019; Veldema & Jansen 2020). Nindorera et al. (2021) reported that overground walking training significantly improved walking endurance in the chronic stroke phase. Nascimento et al. (2021) reported that treadmill training had an equal or superior effect on walking speed and distance in ambulatory people after a stroke. Given that the recovery of mobility after stroke remains the main goal for stroke survivors and a challenge for stroke rehabilitation clinicians (Balasubramanian, Clark & Fox 2014), adding aerobic exercises to conventional care could promote functional recovery of mobility in stroke survivors (MacKay-Lyons et al. 2020).

Our meta-analysis results indicated that mixed AT and RT were more effective for improving QoL in the chronic stroke phase. A previous meta-analysis showed that exercise might have a small to moderate effect on QoL in stroke survivors (Chen & Rimmer 2011). Pang et al. (2013) reported that the efficacy of aerobic exercise in improving QoL was inconclusive. Ali et al. (2021) reported that exercise, including RT, appeared most effective for enhancing QoL’s physical and mental health domains.

Our meta-analysis showed that a higher dosage of AT in time (≥ 120 min per week) and in intensity (≥ 60% HRR or RPE > 14/20) was more effective in improving walking capacity in the chronic stroke phase. The intervention length of the included studies that performed high-dose AT was at least 12 weeks (12–24 weeks), except for one (Ada et al. 2003). Nindorera et al. (2021) reported that an exercise programme including treadmill and overground walking executed at least three times a week, 30 min per session for 8 weeks of intervention, improves walking performance. Luo et al. (2019) reported that a high-intensity exercise programme (70% – 85% HRR/VO2 peak, 3–5 times lasting 30–40 minutes per week for 8–12 weeks) was beneficial for walking competency in patients with subacute and chronic stroke. The body of literature reported that the benefits of AT result from the interaction between the frequency of sessions, session duration and intervention length (MacKay-Lyons et al. 2020). Our review showed that the dosage of these parameters (frequency, intensity and time) is essential in AT interventions to promote walking recovery in the chronic stroke phase.

Our analyses also highlighted that AT programmes executed in hospital settings improved walking capacity in chronic stroke patients. A recent meta-analysis reported insufficient evidence that home-based rehabilitation with usual care might have a short-term effect on stroke survivors’ ability to do basic daily living activities (Qin et al. 2022). The latter meta-analysis did not include trials that implemented structured exercises like those involved in our meta-analysis. Would ongoing supervision of sessions in the hospital setting by practitioners provide additional motivation for stroke survivors? Future studies comparing the implementation of structured exercises in the hospital, at home, or in the community setting would allow us to draw more relevant conclusions.

### Study strengths and limits

Our study provides an updated review of the current evidence related to the use of exercise training protocols and optimal dosage to improve functioning in patients with chronic stroke. So far, meta-analysis of our study is the first to explore the effect of setting on different intervention types. Finally, our review strictly focused on structured exercise programmes for AT and RT interventions. This strategy prevents any parallel effects that may arise from the other means of rehabilitation or other programmes.

However, our results have some potential limitations. Firstly, only one study was maintained in which the impact of exercise on acute or sub-acute stroke was studied. As a result, the stratification of the studies according to the stages of stroke planned in our protocol could not be carried out. Secondly, the modality of exercise (e.g. treadmill, cycling, overground) was not assessed. It is plausible that the modality of exercise could impact the outcomes of interest, especially balance and walking capacity.

## Conclusion

Our review and meta-analysis demonstrated that AT interventions with a higher dosage were most effective in improving walking capacity, and mixed AT and RT was more effective for improving QoL in chronic stroke. However, no superior effect was found with AT and RT programmes on balance compared with control interventions. Hospital-located interventions were more effective on walking capacity than in-home and/or community and laboratory settings.

## Figures and Tables

**FIGURE 1-A1:**
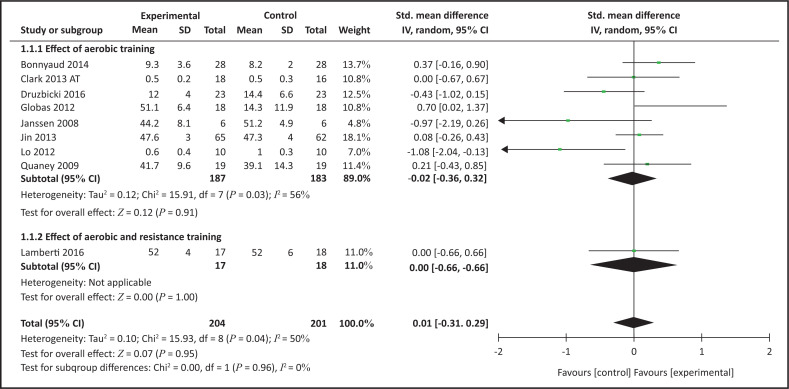
Effect of exercise modes on balance.
